# COVID‐19 vaccine effectiveness by HIV status and history of injection drug use: a test‐negative analysis

**DOI:** 10.1002/jia2.26178

**Published:** 2023-10-26

**Authors:** Joseph H. Puyat, James Wilton, Adeleke Fowokan, Naveed Zafar Janjua, Jason Wong, Troy Grennan, Catharine Chambers, Abigail Kroch, Cecilia T. Costiniuk, Curtis L. Cooper, Darren Lauscher, Monte Strong, Ann N. Burchell, Aslam Anis, Hasina Samji, COVAXHIV Study Team

**Affiliations:** ^1^ British Columbia Centre for Disease Control Vancouver British Columbia Canada; ^2^ School of Population and Public Health University of British Columbia Vancouver British Columbia Canada; ^3^ Centre for Advancing Health Outcomes St Paul's Hospital Vancouver British Columbia Canada; ^4^ Dalla Lana School of Public Health University of Toronto Toronto Ontario Canada; ^5^ The Ontario HIV Treatment Network Toronto Ontario Canada; ^6^ Division of Infectious Diseases and Chronic Viral Illness Service Department of Medicine McGill University Health Centre Montreal Quebec Canada; ^7^ Department of Medicine University of Ottawa Ottawa Ontario Canada; ^8^ CIHR Canadian HIV Trials Network Vancouver British Columbia Canada; ^9^ Pacific AIDS Network Vancouver British Columbia Canada; ^10^ Department of Family and Community Medicine, Faculty of Medicine University of Toronto Toronto Ontario Canada; ^11^ MAP Centre for Urban Health Solutions, Li Ka Shing Knowledge Institute, St. Michael's Hospital Unity Health Toronto Ontario Canada; ^12^ Faculty of Health Sciences Simon Fraser University Burnaby British Columbia Canada

**Keywords:** COVID‐19, SARS‐CoV‐2, vaccine effectiveness, people who use injection drugs, HIV infection, Canada

## Abstract

**Introduction:**

People living with HIV (PLWH) and/or who inject drugs may experience lower vaccine effectiveness (VE) against SARS‐CoV‐2 infection.

**Methods:**

A validated algorithm was applied to population‐based, linked administrative datasets in the British Columbia COVID‐19 Cohort (BCC19C) to ascertain HIV status and create a population of PLWH and matched HIV‐negative individuals. The study population was limited to individuals who received an RT‐PCR laboratory test for SARS‐CoV‐2 between 15 December 2020 and 21 November 2021 in BC, Canada. Any history of injection drug use (IDU) was ascertained using a validated administrative algorithm. We used a test‐negative study design (modified case−control analysis) and multivariable logistic regression to estimate adjusted VE by HIV status and history of IDU.

**Results:**

Our analysis included 2700 PLWH and a matched population of 375,043 HIV‐negative individuals, among whom there were 351 and 103,049 SARS‐CoV‐2 cases, respectively. The proportion of people with IDU history was much higher among PLWH compared to HIV‐negative individuals (40.7% vs. 4.3%). Overall VE during the first 6 months after second dose was lower among PLWH with IDU history (65.8%, 95% CI = 43.5–79.3) than PLWH with no IDU history (80.3%, 95% CI = 62.7–89.6), and VE was particularly low at 4–6 months (42.4%, 95% CI = −17.8 to 71.8 with IDU history vs. 64.0%; 95% CI = 15.7–84.7 without), although confidence intervals were wide. In contrast, overall VE was 88.6% (95% CI = 88.2–89.0) in the matched HIV‐negative population with no history of IDU and remained relatively high at 4–6 months after second dose (84.6%, 95% CI = 83.8–85.4). Despite different patterns of vaccine protection by HIV status and IDU history, peak estimates were similar (≥88%) across all populations.

**Conclusions:**

PLWH with a history of IDU may experience lower VE against COVID‐19 infection, although findings were limited by a small sample size. The lower VE at 4–6 months may have implications for booster dose prioritization for PLWH and people who inject drugs. The immunocompromising effect of HIV, substance use and/or co‐occurring comorbidities may partly explain these findings.

## 1 INTRODUCTION

People living with HIV (PLWH) and people who inject drugs (PWID) are two populations that often overlap and may experience lower vaccine effectiveness (VE) against COVID‐19 infection. Injection drug use (IDU) has been a prominent route of HIV acquisition in British Columbia (BC), Canada and approximately 40% of the PLWH in the province reported a history of IDU at diagnosis [1]. Both PWID and PLWH are socially marginalized populations—contributing to worse socio‐economic status and social determinants of health and consequently a higher burden of COVID‐19 and other illnesses. In addition to a higher burden of potentially immunocompromising comorbidities, HIV infection and substance use can directly impact immune function and may contribute to lower VE in these populations [2, 3, 4, 5, 6].

A VE study recently published by our team suggested that PLWH experience slower development and faster waning of COVID‐19 vaccine protection compared to people not living with HIV, after adjustment for comorbidity burden and other factors [7]. However, the analysis did not explore factors driving the lower VE and/or whether the lower VE was limited to PLWH with specific characteristics. Given the high proportion of PLWH in BC who have a history of IDU—and previous research suggesting VE may be impaired among people who use substances [4]—the objective of this study was to build upon our previous analysis and assess the extent to which HIV and IDU history may jointly impact VE against SARS‐CoV‐2 infection.

## 2 METHODS

### 2.1 Data sources and study population

We adapted and applied a validated algorithm [8] to health services administrative datasets (physician visits, hospitalizations and emergency department visits) [9, 10, 11] in the BC COVID‐19 Cohort (BCC19C) to create a population of PLWH and matched HIV‐negative individuals. The BCC19C is a surveillance platform integrating COVID‐19 datasets with a range of administrative and registry datasets [7]. The HIV‐negative population was created by matching each individual in the PLWH population one‐to‐many to HIV‐negative individuals on the following variables: age (5‐year intervals), sex, community health service area [12] and binary case status (SARS‐CoV‐2 test‐positive vs. test‐negative). HIV‐negative individuals were those who were not flagged by the validated algorithm. Demographic information was extracted from the client roster registry of all individuals enrolled in BC's universal public health insurance programme [13].

The algorithm used to ascertain any history of IDU was based on diagnostic ICD codes for potentially injectable drugs (e.g. excludes alcohol, marijuana and solvent use) and applied to physician visit, hospitalization and emergency department data [9, 10, 11]. The algorithm was based on the full history of available health records and has previously been found to have a sensitivity of 79% and specificity of 82% for accurately ascertaining IDU history [14].

The analysis was limited to individuals who tested for SARS‐CoV‐2 between 15 December 2020 and 21 November 2021. The study period was chosen to coincide with the vaccine rollout in the general population in BC (first dose became available on 15 December) and end prior to the emergence of Omicron.

More detailed information about our data source and methods are outlined in a previous publication [7].

### 2.2 Statistical analysis

A modified case−control approach known as the test‐negative design was used to estimate VE [15]. VE in this context refers to the extent to which COVID‐19 vaccines reduced the risk of SARS‐CoV‐2 infection. The test‐negative design estimates VE by comparing the odds of being a case (binary variable—test‐positive cases vs. test‐negative controls) by vaccination status in a logistic regression analysis. Individuals in the study were classified as a “case” if they tested positive for SARS‐CoV‐2 during the study period (test‐positive) and as a “control” if they tested negative (test‐negative). The composite vaccination status variable used in the logistic regression model to compare odds of being test‐positive was based on number of doses and time since last dose as follows: unvaccinated; ≥14 days after first dose; and 7–59, 60–89, 90–119 and 120–179 days after second dose. First, we described the characteristics of the study population by case status (test‐positive vs. test‐negative), HIV status and history of IDU. Second, a multivariable logistic regression model was conducted with case status (test‐positive vs. test‐negative) as the dependent variable and a 3‐variable interaction term between IDU history, vaccination status and HIV status as the independent variable of primary interest. The regression model was adjusted for the following covariates: age (10‐year age bands), sex, geographic‐level income, health administrative region [12], number of COVID‐19 tests 3 months prior to the study period, Elixhauser comorbidity index (a validated index that sums the presence of 31 comorbidities) [16] and bi‐weekly testing periods. VE was computed using the formula (1−OR) × 100% using the OR estimates from the composite vaccination status variable.

### 2.3 Ethical approval

This study was performed using de‐identified data routinely collected as part of public health surveillance and/or routine healthcare encounters. Patient consent was not required in accordance with the Canadian Tri‐Council Policy Statement: Ethical Conduct for Research Involving Humans article 5.5B. This study was reviewed and approved by the University of British Columbia Research Ethics Board (#H20‐02097).

## 3 RESULTS

### 3.1 Study population

Our analysis included 2700 PLWH and a matched HIV‐negative population of 375,043 HIV‐negative individuals. There were 351 SARS‐CoV‐2 cases among PLWH and 103,049 among HIV‐negative individuals. The proportion of those with any IDU history was much higher among PLWH compared to HIV‐negative individuals (40.7% vs. 4.3%).

Participant characteristics by HIV status, case status, and IDU history are shown in Table 1. Regardless of HIV status, people with a history of IDU had a higher proportion of people with comorbidities and who were living in lower neighbourhood income areas. Of the four study populations in our analysis, people with both HIV and IDU history had the greatest comorbidity burden and neighbourhood income deprivation.

**Table 1:** Characteristics of individuals tested for SARS‐CoV‐2 between 15 December 2020 and 21 November 2021 by HIV status, SARS‐CoV‐2 case status and history of injection drug use in British Columbia, Canada

	PLWH (*n* = 2700)				Matched HIV‐negative population (*n* = 375,043)			
	IDU history (*n* = 1098)		No IDU history (*n* = 1602)		IDU history (*n* = 16,260)		No IDU history (*n* = 358,783)	
	Case (*n* = 176) *n* (%)	Control (*n* = 922) *n* (%)	Case (*n* = 175) *n* (%)	Control (*n* = 1427) *n* (%)	Case (*n* = 5703) *n* (%)	Control (*n* = 10,557) *n* (%)	Case (*n* = 97,346) *n* (%)	Control (*n* = 261,437) *n* (%)
**Sex—Female**	74 (42.0)	319 (34.6)	53 (30.3)	327 (22.9)	2481 (43.5)	4512 (42.7)	48,873 (50.2)	134,207 (51.3)
**Age**								
Median (SD)	47 (15.0)	50 (15.0)	51 (18.0)	53 (21.0)	38 (20.0)	42 (25.0)	43 (26.0)	51 (32.0)
19–29	<10	51 (5.5)	11 (6.3)	95 (6.7)	1574 (27.6)	2620 (24.8)	21,873 (22.5)	41,117 (15.7)
30–39	39 (22.2)	163 (17.7)	29 (16.6)	230 (16.1)	1545 (27.1)	2205 (20.9)	20,712 (21.3)	42,136 (16.1)
40–49	57 (32.4)	235 (25.5)	40 (22.9)	262 (18.4)	1166 (20.4)	2072 (19.6)	17,932 (18.4)	40,742 (15.6)
50–59	49 (27.8)	337 (36.6)	51 (29.1)	392 (27.5)	919 (16.1)	2013 (19.1)	15,891 (16.3)	41,026 (15.7)
60–69	21 (11.9)	131 (14.2)	30 (17.1)	283 (19.8)	449 (7.9)	1425 (13.5)	11,193 (11.5)	40,720 (15.6)
70 and over	<5	5 (0.5)	14 (8.0)	165 (11.6)	50 (0.9)	222 (2.1)	9745 (10.0)	55,696 (21.3)
**Elixhauser Comorbidity Index**								
Median (SD)	5.5 (4.0)	5 (4.0)	2 (2.0)	2 (3.0)	3 (3.0)	4 (4.0)	1 (2.0)	2 (2.0)
0	<5	9 (1.0)	39 (22.3)	259 (18.1)	295 (5.2)	481 (4.6)	32,138 (33.0)	63,836 (24.4)
1	<5	35 (3.8)	34 (19.4)	307 (21.5)	687 (12.0)	966 (9.2)	25,035 (25.7)	59,777 (22.9)
2	12 (6.8)	77 (8.4)	46 (26.3)	262 (18.4)	866 (15.2)	1474 (14.0)	16,116 (16.6)	45,565 (17.4)
3 or more	161 (91.5)	801 (86.9)	56 (32.0)	599 (42.0)	3855 (67.6)	7636 (72.3)	24,057 (24.7)	92,259 (35.3)
**Number of tests 3 months before 15 December 2020**								
0	110 (62.5)	583 (63.2)	145 (82.9)	1124 (78.8)	4478 (78.5)	8229 (77.9)	83,627 (85.9)	218,338 (83.5)
1	46 (26.1)	214 (23.2)	24 (13.7)	241 (16.9)	935 (16.4)	1821 (17.2)	11,891 (12.2)	36,473 (14.0)
2	11 (6.3)	73 (7.9)	<5	51 (3.6)	208 (3.6)	348 (3.3)	1365 (1.4)	5047 (1.9)
3 or more	9 (5.1)	52 (5.6)	<5	11 (0.8)	82 (1.4)	159 (1.5)	463 (0.5)	1579 (0.6)
**Health authority**								
Interior	13 (7.4)	66 (7.2)	10 (5.7)	123 (8.6)	949 (16.6)	1903 (18.0)	18,097 (18.6)	52,621 (20.1)
Fraser	45 (25.6)	183 (19.8)	66 (37.7)	468 (32.8)	1855 (32.5)	2580 (24.4)	40,320 (41.4)	68,001 (26.0)
Vancouver Coastal	95 (54.0)	561 (60.8)	80 (45.7)	657 (46.0)	1362 (23.9)	2382 (22.6)	21,402 (22.0)	60,631 (23.2)
Vancouver Island	13 (7.4)	67 (7.3)	7 (4.0)	150 (10.5)	889 (15.6)	2441 (23.1)	7188 (7.4)	55,099 (21.1)
Northern	10 (5.7)	43 (4.7)	12 (6.9)	28 (2.0)	643 (11.3)	1238 (11.7)	10,155 (10.4)	24,738 (9.5)
Missing	<5	<5	<5	<5	5 (0.1)	13 (0.1)	184 (0.2)	347 (0.1)
**Neighbourhood income**								
Lowest quintile	96 (54.5)	487 (52.8)	61 (34.9)	484 (33.9)	2262 (39.7)	3550 (33.6)	21,369 (22.0)	51,412 (19.7)
2	22 (12.5)	141 (15.3)	38 (21.7)	320 (22.4)	1117 (19.6)	2092 (19.8)	20,026 (20.6)	49,534 (18.9)
3	27 (15.3)	140 (15.2)	30 (17.1)	281 (19.7)	887 (15.6)	1834 (17.4)	19,283 (19.8)	53,634 (20.5)
4	23 (13.1)	102 (11.1)	28 (16.0)	217 (15.2)	880 (15.4)	1799 (17.0)	19,509 (20.0)	54,815 (21.0)
Highest quintile	8 (4.5)	50 (5.4)	18 (10.3)	124 (8.7)	551 (9.7)	1265 (12.0)	16,971 (17.4)	51,663 (19.8)
Missing	<5	<5	<5	<5	6 (0.1)	17 (0.2)	188 (0.2)	379 (0.1)
**Vaccination status**								
Unvaccinated	130 (73.9)	686 (74.4)	145 (82.9)	989 (69.3)	4766 (83.6)	7832 (74.2)	81,845 (84.1)	171,375 (65.6)
First dose (≥14 days)	9 (5.1)	41 (4.4)	<5	13 (0.9)	200 (3.5)	267 (2.5)	1082 (1.1)	2639 (1.0)
Second dose (days)								
≥7	32 (18.2)	172 (18.7)	25 (14.3)	386 (27.0)	588 (10.3)	2270 (21.5)	13,168 (13.5)	82,656 (31.6)
7–59	<10	46 (5.0)	7 (4.0)	125 (8.8)	138 (2.4)	829 (7.9)	2549 (2.6)	23,823 (9.1)
60–89	<5	42 (4.6)	<5	75 (5.3)	132 (2.3)	525 (5.0)	3077 (3.2)	20,212 (7.7)
90–119	<10	39 (4.2)	<5	99 (6.9)	142 (2.5)	494 (4.7)	3439 (3.5)	20,296 (7.8)
120–179	13 (7.4)	40 (4.3)	9 (5.1)	81 (5.7)	142 (2.5)	367 (3.5)	3310 (3.4)	16,614 (6.4)

*Note*: Percentages are column percentages. Small counts were suppressed as per data steward requirements.

Abbreviations: IDU, injection drug use; PLWH, people living with HIV; SD, standard deviation.

### 3.2 VE estimates

**Figure 1:**
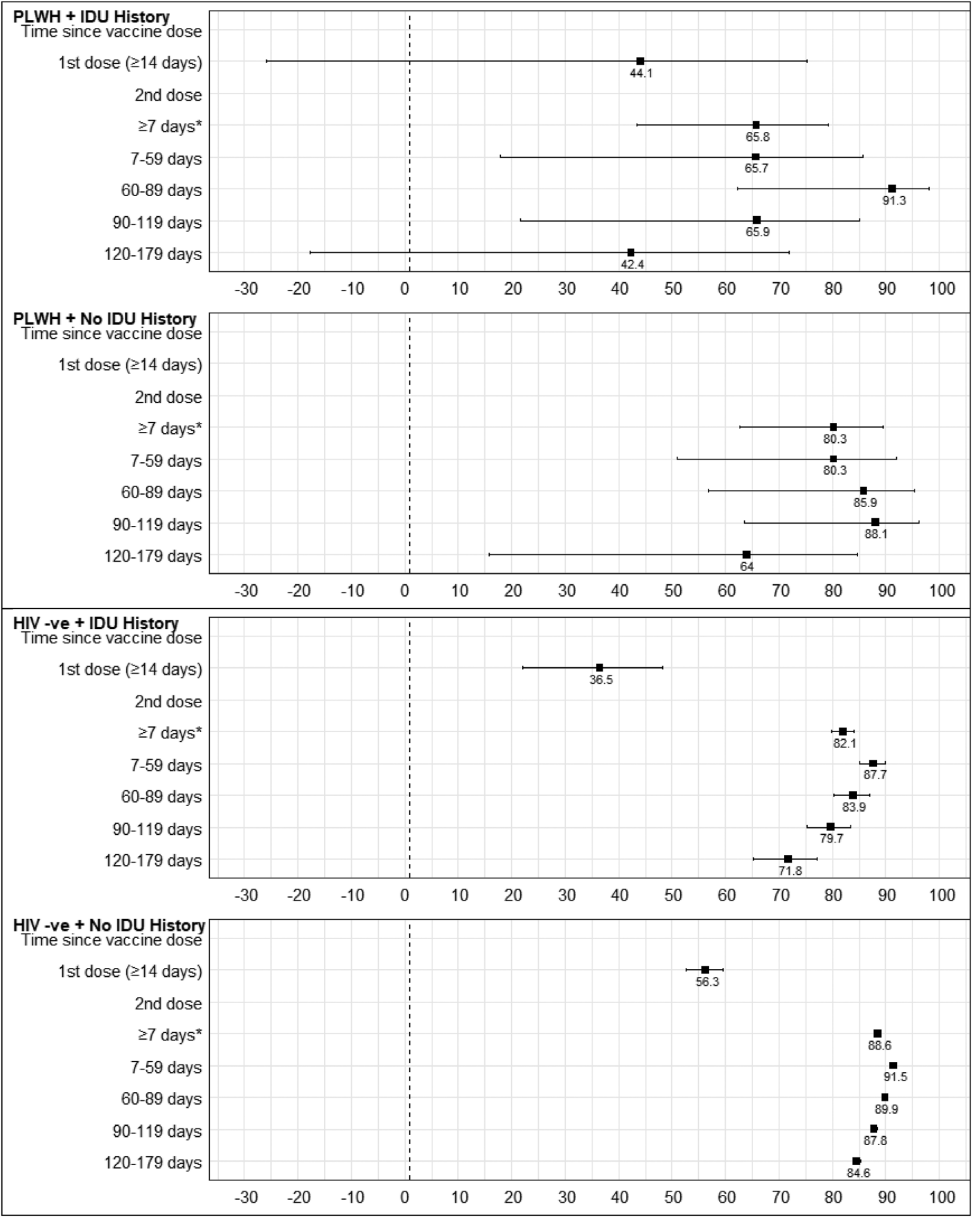
Adjusted vaccine effectiveness estimates by HIV status and history of injection drug use among individuals tested for SARS‐CoV‐2 between 15 December 2020 and 21 November 2021 in British Columbia, Canada. IDU, injection drug use; PLWH, people living with HIV. * Modelled separately.

VE estimates by HIV status and history of IDU are presented in Table 2 and Figure 1. Lower VE estimates were generally observed for PLWH (vs. HIV‐negative individuals) and for people with a history of IDU (vs. no history of IDU). These differences were particularly pronounced among PLWH with a history of IDU—the population with the lowest VE overall and at 4–6 months—although the sample size in this population was the smallest, leading to variability in estimates, wide confidence intervals and potential statistical non‐significance for comparisons. Overall VE estimates during first 6 months of second dose were lowest among PLWH with IDU history (65.8%, 95% CI = 43.5–79.3), similar for PLWH with no IDU history (80.3%, 95% CI = 62.7–89.6) and HIV‐negative individuals with IDU history (82.1%, 95% CI = 79.9–84.1) and highest for HIV‐negative individuals with no IDU history (88.6%, 95% CI = 88.2–89.0). Estimates at 4–6 months were also lowest among PLWH with history of IDU (42.4%, 95% CI = −17.8 to 71.8), followed by PLWH with no history of IDU (64.0%, 95% CI = 15.7–84.7), HIV negative with history of IDU (71.8%, 95% CI = 65.2–77.1) and HIV negative with no history of IDU (84.6%, 95% CI = 83.8–85.4).

**Table 2:** Adjusted vaccine effectiveness estimates by HIV status and history of injection drug use among individuals tested for SARS‐CoV‐2 between 15 December 2020 and 21 November 2021 in British Columbia, Canada

	PLWH						Matched HIV‐negative population					
	IDU history			No IDU history			IDU history			No IDU history		
	Estimate	LCL	UCL	Estimate	LCL	UCL	Estimate	LCL	UCL	Estimate	LCL	UCL
First dose (≥14 days)	44.1	−25.8	75.2	40.7	−198.8	88.2	36.5	22.1	48.2	56.3	52.7	59.6
Second dose												
≥7 days*	65.8	43.5	79.3	80.3	62.7	89.6	82.1	79.9	84.1	88.6	88.2	89.0
7–59	65.7	17.9	85.7	80.3	50.9	92.1	87.7	85.1	89.9	91.5	91.1	92.0
60–89	91.3	62.3	98.0	85.9	56.8	95.4	83.9	80.3	86.9	89.9	89.4	90.3
90–119	65.9	21.6	85.2	88.1	63.6	96.1	79.7	75.2	83.4	87.8	87.2	88.4
120–179	42.4	−17.8	71.8	64.0	15.7	84.7	71.8	65.2	77.1	84.6	83.8	85.4

*Note*: Adjusted VE estimates derived from a 3‐variable interaction term (vaccination X PLWH X IDU history) in a multivariable logistic regression model with case status as dependent variable. All results (except for row marked by *) are derived from a single model with the following categorization for vaccination status: unvaccinated, first dose (≥14 days), second dose (7–59 days), second dose (60–89 days), second dose (90–119 days) and second dose (120–179 days). * Indicates results derived from a separate model with vaccination status categorized as unvaccinated and second dose (≥7 days). Days refer to the number of days elapsed between dose and date of SARS‐CoV‐2 RT‐PCR test. Lightly shaded values have extremely wide confidence intervals and were therefore shown on Table 2 only and suppressed in Figure 1.

Abbreviations: LCL, lower confidence limit; PLWH, people living with HIV; UCL, upper confidence limit.

## 4 DISCUSSION

Our analysis suggests PLWH with a history of IDU may experience lower VE against laboratory‐confirmed SARS‐CoV‐2 infection. Overall VE during the first 6 months of second dose was lowest—and the degree of waning at 4–6 months greatest—among PLWH with a history of IDU compared to other populations included in our analysis (PLWH with no IDU history, matched HIV‐negative individuals with or without a history of IDU). Of note, the sample size of PLWH with a history of IDU was also the smallest, leading to wide confidence intervals and an inability to make definitive conclusions. Notably, VE was also lower in the large sample of matched HIV‐negative people with a history of IDU in our analysis (vs. HIV negative with no history of IDU), and our results are supported by other studies suggesting that COVID‐19 VE may be lower among people who use substances [4]. The results also suggest our previous finding of slower buildup/faster waning of VE among PLWH overall (vs. HIV‐negative overall) [7] may be partly related to the impact of substance use—and/or comorbidities that co‐occur with IDU—on immune function. Indeed, the proportion of people with IDU history was almost 10 times higher among PLWH in our analysis (vs. HIV‐negative individuals; 40.7% vs. 4.3%) and people with HIV and/or IDU history were also more likely to have other comorbidities. These findings may have implications for booster dose prioritization for PLWH and PWID.

Our results could be due to a direct impact of substance use on the immune system (although we could not determine whether people were currently injecting and/or using substances) [2], the impact of immunocompromising comorbidities that co‐occur with IDU [5, 6] and/or other factors that may impact immune function (e.g. HIV treatment adherence). In addition to biological explanations, differences may be due to other factors related to social determinants of health and possible exposures (e.g. communal housing and challenges with physical distancing) [17, 18]. In a large US‐based study, a higher risk of breakthrough infection was identified among people with substance use history, but this finding was no longer present after propensity‐score matching on certain comorbidities and adverse social determinants of health (except for people with cannabis dependence) [4]. In our analysis, we adjusted for the number of comorbidities using the Elixhauser comorbidity index, which may have led to some residual confounding. Matching on specific comorbidities in our analysis was not possible due to the relatively small sample size and number of outcomes in our PLWH population. Future analyses will focus on a general population sample and include more rigorous adjustment for comorbidities, in addition to stratification by active versus past IDU and type of substance injected.

Regardless of the potential for residual confounding, our results (and results from other studies) suggest people with HIV and/or a history of IDU may benefit from booster dose prioritization given the lower estimates of second dose VE at 4–6 months in these populations. Our results may also highlight how intersecting health and social conditions can reinforce each other to increase vulnerability to negative health outcomes, as evidenced by the lowest VE being observed among people with both HIV and IDU history. Promisingly, however, significant protection against SARS‐CoV‐2 infection was still afforded—and peak level of vaccine protection was generally comparable—across populations. Vaccine prioritization among PLWH and PWID may require additional effort to overcome the unique barriers to COVID‐19 vaccine uptake faced by these populations (e.g. stigma and discrimination, medical mistrust), as identified in other studies [19].

In addition to the potential for residual confounding, our analysis had other limitations, including a small sample size for the PLWH analysis and potential misclassification due to the use of administrative algorithms to ascertain HIV and the history of IDU. Further, we did not differentiate between active versus past IDU, due to the small sample size, limiting the generalizability of study findings. We also lacked clinical information on PLWH, including CD4 count and HIV viral load.

## 5 CONCLUSIONS

In conclusion, these results build upon our previous paper by suggesting the lower VE previously observed among PLWH may be partly related to the impact of substance use and/or immunocompromising health conditions that can co‐occur with IDU. This is supported by our finding of lower VE among PLWH who have a history of IDU, as well as other studies suggesting that VE may be lower among people who use substances. These findings may have implications for low‐barrier COVID‐19 vaccine booster prioritization efforts.

## COMPETING INTERESTS

The authors declare no competing interests.

## AUTHORS’ CONTRIBUTIONS

JHP: Investigation, formal analysis, methodology, writing—review & editing. JW: Writing—original draft, methodology. AF: Writing—review & editing, visualization, project administration, methodology. HS: Funding acquisition, project administration, supervision, writing—review & editing, methodology, visualization. NZJ: Conceptualization, funding acquisition, writing—review & editing, visualization. ANB and AA: Conceptualization, funding acquisition, writing—review & editing. DL, MS, AK, CC, CTC, CLC and TG: Writing—review & editing. All authors approved the manuscript.

## FUNDING

This project is being supported by funding from the Public Health Agency of Canada 2122‐HQ‐000075, through the Vaccine Surveillance Reference group and the COVID‐19 Immunity Task Force and the Canadian Institutes for Health Research Canadian HIV Trials Network. JHP is supported by the Michael Smith Health Research BC (Scholar Awards). ANB is a Canada Research Chair in Sexually Transmitted Infection Prevention; she is also a recipient of a Non‐Clinician Scientist Award from the Department of Family and Community Medicine, Faculty of Medicine, University of Toronto. CTC holds a chercheur‐boursier clinicien Fonds de recherche du Québec‐Santé Junior 2 award.

## DISCLAIMER

All inferences, opinions and conclusions drawn in this manuscript are those of the authors, and do not reflect the opinions or policies of the Data Steward(s).

## Data Availability

The data that support the findings of this study are available on request from the corresponding author. The data are not publicly available due to privacy or ethical restrictions.

## References

[1] Heath K , Samji H , Nosyk B , Colley G , Gilbert M , Hogg RS , et al. Cohort profile: seek and treat for the optimal prevention of HIV/AIDS in British Columbia (STOP HIV/AIDS BC). Int J Epidemiol. 2014; 43(4):1073–1081. 24695113 10.1093/ije/dyu070 PMC4121558

[2] Friedman H , Newton C , Klein TW. Microbial infections, immunomodulation, and drugs of abuse. Clin Microbiol Rev. 2003; 16(2):209–219. 12692094 10.1128/CMR.16.2.209-219.2003 PMC153143

[3] Yin J , Chen Y , Li Y , Wang C , Zhang X. Immunogenicity and efficacy of COVID‐19 vaccines in people living with HIV: a systematic review and meta‐analysis. Int J Infect Dis. 2022; 124:212–223. 36241168 10.1016/j.ijid.2022.10.005 PMC9553964

[4] Wang L , Wang Q , Davis PB , Volkow ND , Xu R. Increased risk for COVID‐19 breakthrough infection in fully vaccinated patients with substance use disorders in the United States between December 2020 and August 2021. World Psychiatry. 2022; 21(1):124–132. 34612005 10.1002/wps.20921 PMC8661963

[5] Lerner AM , Eisinger RW , Fauci AS. Comorbidities in persons with HIV: the lingering challenge. JAMA. 2020; 323(1):19. 31825458 10.1001/jama.2019.19775

[6] Common comorbidities with substance use disorders research report [Internet]. Bethesda, MD: National Institutes on Drug Abuse (US); 2020 [cited 2023 Feb 6]. Available from: http://www.ncbi.nlm.nih.gov/books/NBK571451/ 34185444

[7] Fowokan A , Samji H , Puyat JH , Janjua NZ , Wilton J , Wong J , et al. Effectiveness of COVID‐19 vaccines in people living with HIV in British Columbia and comparisons with a matched HIV‐negative cohort: a test‐negative design. Int J Infect Dis. 2022; 127:162–170. 36462571 10.1016/j.ijid.2022.11.035 PMC9711901

[8] Nosyk B , Colley G , Yip B , Chan K , Heath K , Lima VD , et al. Application and validation of case‐finding algorithms for identifying individuals with human immunodeficiency virus from administrative data in British Columbia, Canada. PLoS ONE. 2013;8(1):e54416. 23382898 10.1371/journal.pone.0054416 PMC3557280

[9] British Columbia Ministry of Health . Medical Services Plan (MSP) Payment Information File. British Columbia Ministry of Health; 2020. https://www2.gov.bc.ca/gov/content/health/health‐forms/online‐services.

[10] British Columbia Ministry of Health . Discharge Abstract Database (Hospital Separations). British Columbia Ministry of Health; 2020. https://www2.gov.bc.ca/gov/content/health/health‐forms/online‐services.

[11] British Columbia Ministry of Health . National Ambulatory Care Reporting System. British Columbia Ministry of Health; 2020. https://www2.gov.bc.ca/gov/content/health/health‐forms/online‐services.

[12] BC Centre for Disease Control . BC Community Health Service Area Health Profiles (Version 1.0) accessed August 23, 2023 [Internet]. Available from: http://communityhealth.phsa.ca/CHSAHealthProfiles

[13] British Columbia Ministry of Health . Client Roster (Client Registry System/Enterprise Master Patient Index). British Columbia Ministry of Health; 2020. https://www2.gov.bc.ca/gov/content/health/health‐forms/online‐services.

[14] Janjua NZ , Islam N , Kuo M , Yu A , Wong S , Butt ZA , et al. Identifying injection drug use and estimating population size of people who inject drugs using healthcare administrative datasets. Int J Drug Policy. 2018; 55:31–39. 29482150 10.1016/j.drugpo.2018.02.001

[15] Skowronski DM , Masaro C , Kwindt TL , Mak A , Petric M , Li Y , et al. Estimating vaccine effectiveness against laboratory‐confirmed influenza using a sentinel physician network: results from the 2005–2006 season of dual A and B vaccine mismatch in Canada. Vaccine. 2007; 25(15):2842–2851. 17081662 10.1016/j.vaccine.2006.10.002

[16] Elixhauser A , Steiner C , Harris DR , Coffey RM. Comorbidity measures for use with administrative data. Med Care. 1998;36(1):8–27. 9431328 10.1097/00005650-199801000-00004

[17] Xia Y , Ma H , Moloney G , Velásquez García HA , Sirski M , Janjua NZ , et al. Geographic concentration of SARS‐CoV‐2 cases by social determinants of health in metropolitan areas in Canada: a cross‐sectional study. Can Med Assoc J. 2022; 194(6):E195–204. 35165131 10.1503/cmaj.211249 PMC8900797

[18] Menza TW , Hixson LK , Lipira L , Drach L. Social determinants of health and care outcomes among people with HIV in the United States. Open Forum Infect Dis. 2021; 8(7):ofab330. 34307729 10.1093/ofid/ofab330 PMC8297699

[19] Menza TW , Capizzi J , Zlot AI , Barber M , Bush L. COVID‐19 vaccine uptake among people living with HIV. AIDS Behav. 2022; 26(7):2224–2228. 34994913 10.1007/s10461-021-03570-9 PMC8739679

